# Repetitive transcranial magnetic stimulation reveals a causal role of the human precuneus in spatial updating

**DOI:** 10.1038/s41598-018-28487-7

**Published:** 2018-07-05

**Authors:** Notger G. Müller, Martin Riemer, Lisa Brandt, Thomas Wolbers

**Affiliations:** German Centre for Neurodegenerative Diseases (DZNE), Center for Behavioral Brain Sciences (CBBS) & Medical Faculty at Otto von Guericke University, Leipziger Str. 44, 39120 Magdeburg, Germany

## Abstract

As we move through an environment, the positions of surrounding objects relative to our body constantly change, with some objects even leaving our field of view. As a consequence, maintaining orientation requires spatial updating, the continuous monitoring of self-motion cues to update external locations within an egocentric frame of reference. While previous research using functional magnetic resonance imaging has implicated the precuneus in spatial updating, direct evidence for this claim is missing. To address this important question, we applied theta burst repetitive transcranial magnetic stimulation (rTMS) over the precuneus to induce a “virtual lesion”. Following stimulation, participants were tested in a large-scale virtual environment in which they had to use visual self-motion information to keep track of the position of virtual objects. Compared to sham stimulation, rTMS affected working memory traces for object locations. Critically, rTMS further impaired the ability to update these locations whenever participants experienced simulated movement. As this effect could not be explained by working memory deficits alone, we conclude that visual spatial updating relies on the construction of updated representations of egocentric object locations within the precuneus. Together, these findings establish the precuneus as performing key computations for the formation of cognitive maps.

## Introduction

Imagine the situation when you walk or drive through an unknown city: Some objects, e.g. the bank building in front of you, “move” nearer whilst others, e.g. the pharmacy on your left “move” away until they get out of your field of view. Nevertheless when you are asked by a passerby where to find the next pharmacy you are able to point to the correct location. And this is even the case, when you are not simply moving straight forward but when you make turns and move at different speeds. The process of calculating new relative positions of surrounding objects with respect to oneself (also referred to as egocentric space) during self-motion is referred to as spatial updating.

In search of the neural mechanisms supporting spatial updating, Wolbers *et al*.^1^ conducted an fMRI study in which self-motion was simulated with an expanding optic flow field. Before the onset of optic flow subjects were briefly presented with objects, which disappeared after several seconds. At the end of the simulated self-motion subjects had to indicate the assumed new positions of the vanished objects. In order to identify areas specifically involved in updating of spatial information during self-motion two manipulations were applied: static (no optic flow) trials were compared to dynamic (with simulated motion) trials, and the number of objects to be memorized varied. The idea here was that a brain region in which memory representations of object locations are updated during self-motion should be sensitive to both the presence of optic flow and working memory load, i.e., the number of locations in egocentric space which need to be updated^2^. Activity in only two brain regions was modulated by both manipulations, namely in the precuneus and in the left dorsal precentral gyrus, respectively. However, the latter showed updating-related brain responses only when the task required a pointing response but not when responses could be given verbally. Hence, the authors concluded that the frontal brain region is involved in the planning of motor actions whereas the parietal precuneus is specifically recruited for spatial updating (see also^3–5^).

While subsequent studies confirmed the importance of the medial parietal lobe for spatial updating^6^, some issues have been unresolved to date. Studies using fMRI provide only limited space within the bore of the MR scanner, and therefore, stimuli are usually presented on small screens^1,6,7^. As a consequence, the induced vection – the feeling of self-motion – is relatively weak. Furthermore, although fMRI studies can suggest causal information about the relationship between neuronal activation patterns and behavioural processes^8^, such a causal link would be strengthened by studies using transcranial magnetic stimulation (TMS). Thus, a TMS study can rule out the possibility that the precuneus was activated simply because it is involved in the automatic preparation of a pointing response, even though the task itself did not require such a response. Indeed, discrepancies between lesion and fMRI studies have been observed repeatedly^9^. For example, whereas fMRI studies have identified the hippocampal formation to be involved in path integration^10,11^, studies in stroke patients have shown that corresponding lesions do not necessarily result in a reduced path integration performance^12^.

To overcome these limitations, the present study adopted an optimized experimental approach. First, we presented the virtual environment on a large-scale display, which is known to evoke a much stronger feeling of vection. In dynamic trials, subjects experienced simulated self-motion after the to-be-memorized objects had disappeared; static trials without motion served as control. Second, we employed repetitive TMS over the precuneus to induce a “virtual lesion” of this brain area (e.g.^13^). Given that inhibitory effects of TMS protocols can change into excitatory effects - depending on the duration of the stimulation^14^ - we applied TMS for 44 s. This continuous theta burst protocol (cTBS) has previously been shown to result in an inhibition of cortical areas^15–17^. In addition, it enabled us to study spatial updating without concomitant TMS (see methods), thus addressing the problem that potential changes in performance could be driven by unspecific stimulation effects^15,18^. We reasoned that if interfering with information processing in the precuneus led to a selective deficit in spatial updating (i.e., a deficit that cannot solely to be explained by disturbed working memory), a crucial role in the construction of updated spatial memories can be postulated.

## Results

**Figure 1:**
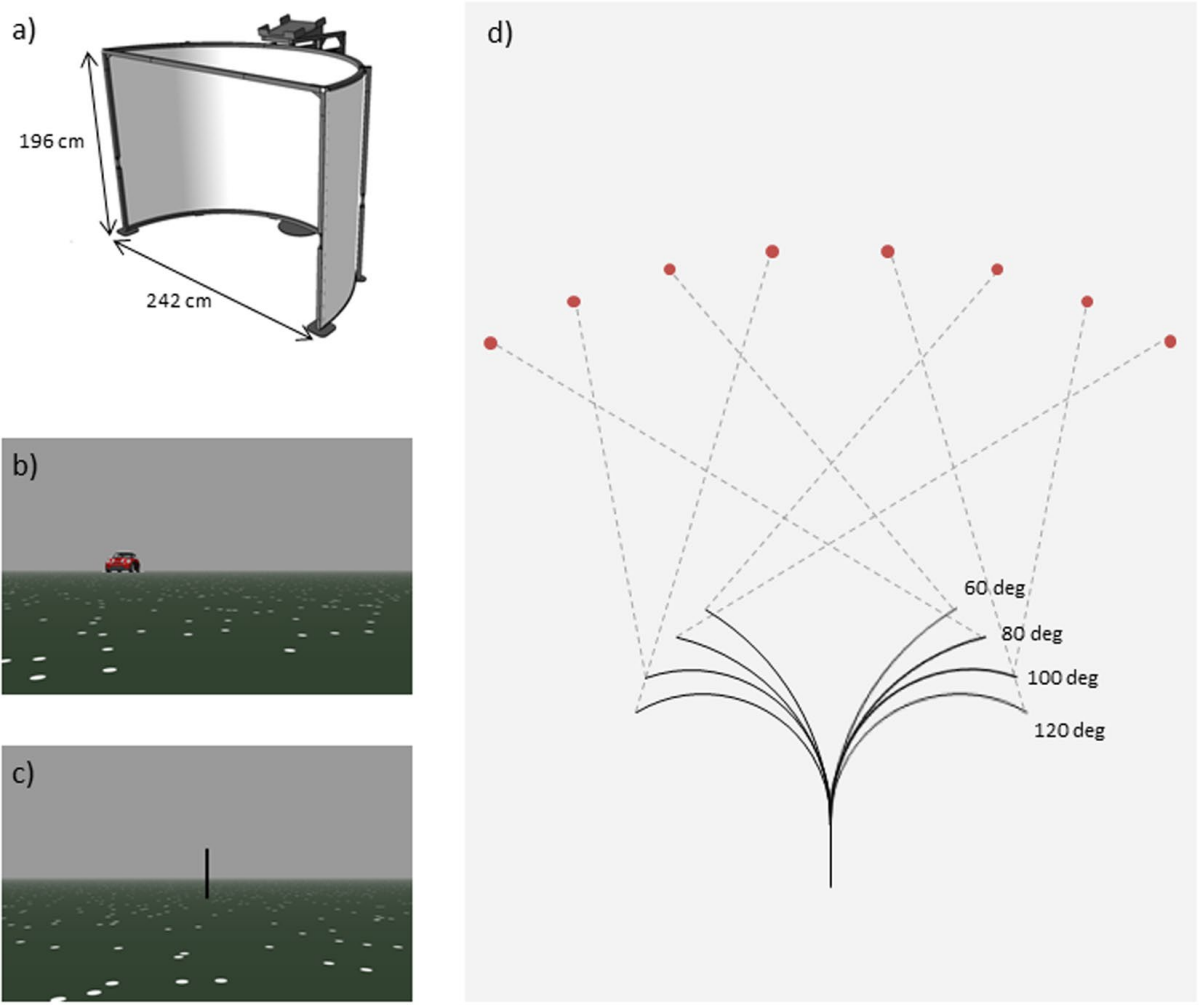
:Experimental setup. (**a**) Circular screen used for the presentation of the virtual environment as shown in (**b**) and (**c**). (**d**) Schematic depiction of the eight different paths and their respective target locations. Numbers in red denote the updating angle associated with each path-target combination. (**e**) Updating angle was quantified by the angular difference of the egocentric direction towards the target object before (green angle) and after passive movement (blue angle). Copyright Information: (**a**) was provided for this publication by the manufacturer of the c-screen (Arene Tech, Strassbourg, France). (**b,c**) are screenshots from the actual experiment. (**d,e**) was drawn by one of the authors (MR).

Participants performed a spatial updating task within a virtual environment where self-motion was simulated in dynamic trials (Fig. 1b,c; cf.^1^). In these trials a target object was presented at one of eight possible locations (Fig. 1b). After the target object vanished the observer was passively moved along one of eight possible paths (cf. Fig 1d). After the movement was completed, participants were asked to rotate with a joystick to until they faced the remembered location of the target object (Fig. 1c). Each path was combined with a specific target location (cf. Fig 1d), so that four path types could be specified, each including both left- and rightward turns. Each path type was associated with a certain updating angle, i.e., the extent to which spatial updating of the target location was required (see the section on Statistical Analysis and Fig. 1e). Updating angle for path type 1 to 4 was 135°, 108°, 117° and 80°, respectively.

**Figure 2:**
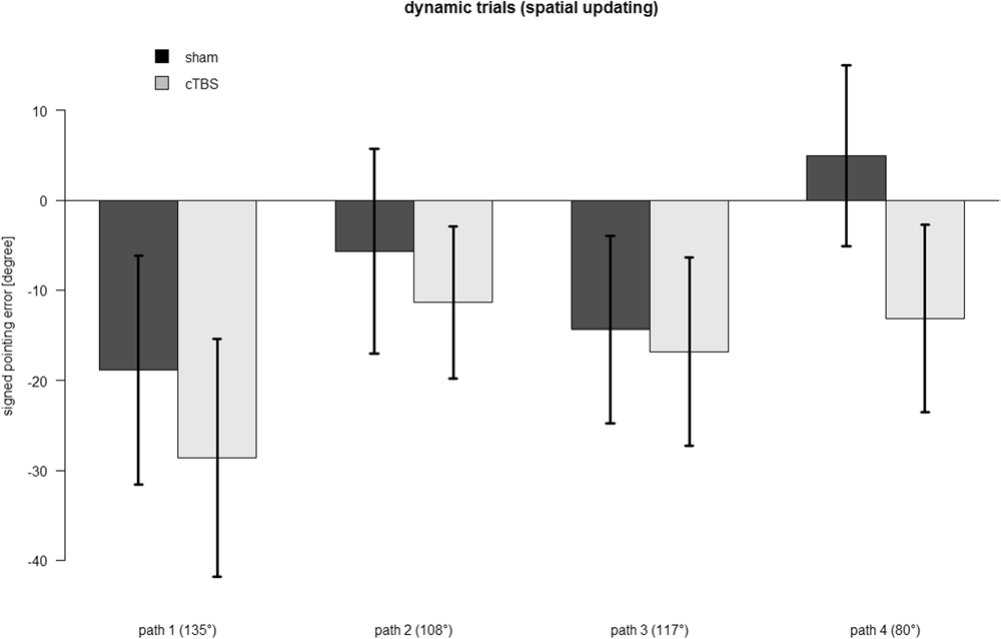
Response accuracy in spatial updating trials. Mean signed pointing error towards the facing direction at the end of the passive movement were significantly increased after inhibition of the precuneus (grey) as compared to sham stimulation (black). Degree values on the x-axis denote the updating angle associated with each path. Error bars show the standard error of the mean across participants.

In dynamic trials (Fig. 2), we observed a signed pointing error, i.e., an undershooting of correct angle, that was significantly larger in the TMS compared to the sham condition (F_1,151_ = 5.5, p = .02, L.Ratio = 5.2). The signed pointing error also differed across the four path types (F_1,151_ = 9.1, p = .003, L.Ratio = 9.0). Importantly, there was no interaction between TMS condition and path type, demonstrating that the effect of TMS was comparable across all four path types (F_1,151_ = 0.4, p = .527, L.Ratio = 0.4). As Fig. 2 indicates a more pronounced pointing error for path types associated with a larger updating angle, we performed post-hoc t-tests between the path types. A larger signed pointing error was indeed found for path type 1 compared to path type 2 (t_43_ = 3.1, p = .002, *d* = 0.28) and for path type 3 compared to path type 4 (t_43_ = 2.6, p = .007, *d* = 0.24). In line with the larger updating angle for path type 3 (117°) compared to path type 2 (108°), a larger signed pointing error was also found for the former (t_43_ = 1.7, p = .046, *d* = 0.15).

**Figure 3:**
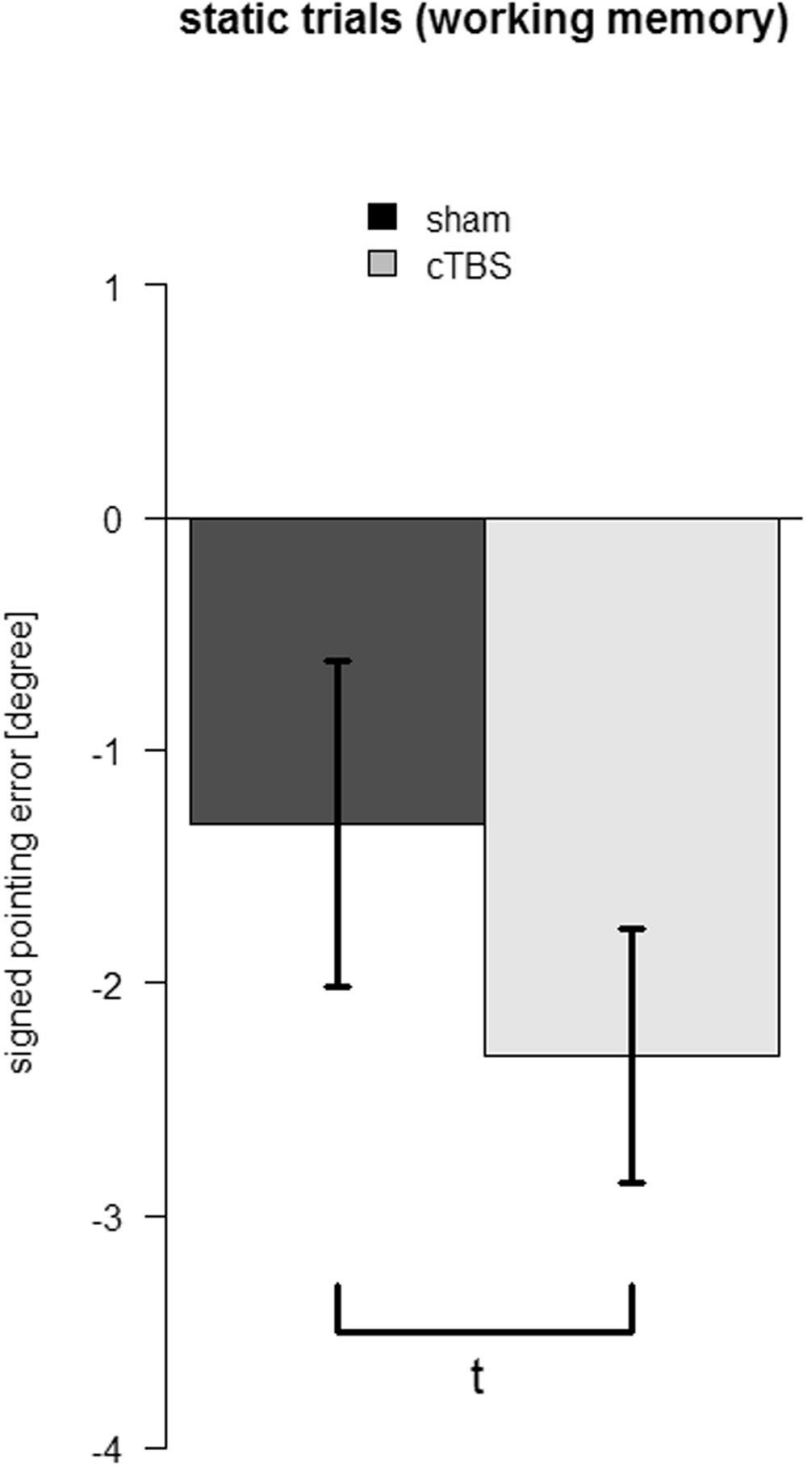
Pointing accuracy in static trials. Mean signed pointing error shows a trend towards a significant increase after inhibition of the precuneus (grey) as compared to sham stimulation (black). Error bars show the standard error of the mean across participants (t: p < .1).

To account for working memory effects independent of spatial updating performance, subjects also performed static trials. In these trials, no optic flow was presented but the remaining procedures were identical to the dynamic trials. Results of static trials are depicted in Fig. 3. Similar to dynamic trials, rTMS resulted in a trend for a larger pointing error towards the facing direction at the end of the passive movement (t_21_ = 1.7, p = .056, *d* = 0.34), rendering it possible that the TMS application interfered with processes related to working memory rather than spatial updating of locations. As the orientation of the observer did not change in static trials and therefore the required turning angle to point at the target object was smaller, the signed pointing error was generally smaller in static as compared to dynamic trials.

To determine whether the variance in dynamic trials could be explained solely on the basis of the variance in static trials, we used a linear regression model to predict the pointing error in dynamic trials from the pointing error observed in static trials. The residuals from this regression model were then entered in another mixed-model analysis. In this analysis, the same pattern of results was observed (TMS condition: F_1,151_ = 10.1, p = .002, L.Ratio = 9.4; path type: F_1,151_ = 8.7, p = .004, L.Ratio = 8.6; TMS condition × path type: F_1,151_ = 0.4, p = .535, L.Ratio = 0.4), showing that the TMS effect on performance in the dynamic trials cannot solely be explained by changes in working memory processes. Another way of testing for a modulatory effect of working memory is to subtract the pointing error in static trials from the pointing error in dynamic trials. An analysis performed on these difference values again revealed the same pattern of results (TMS condition: F_1,151_ = 4.3, p = .039, L.Ratio = 4.1; path type: F_1,151_ = 9.1, p = .003, L.Ratio = 8.9; TMS condition × path type: F_1,151_ = 0.4, p = .528, L.Ratio = 0.4).

To test whether the inhibitory effect of cTBS was effective over the complete period of the experiment, we compared the performance during the first and the last third of trials. As in the main analysis, we found a main effect of TMS condition (F_1,323_ = 4.3, p = .038, L.Ratio = 4.1) and of path type (F_1,323_ = 13.3, p < .001, L.Ratio = 13.0). The main effect of first vs. last third of trials was significant as well (F_1,323_ = 7.3, p = .007, L.Ratio = 7.4), indicating that the participants were more accurate in later trials. Importantly, this effect did not interact with TMS condition (F_1,323_ < 0.1, p = .947, L.Ratio < 0.1), demonstrating that the effect of TMS was not different between the first and the last trials.

In addition to affecting the signed pointing error, we also tested whether TMS led to noisier representations of the object positions. To quantify noise, we calculated the variability of the pointing responses across replications of a given path type. In contrast to the results from the pointing error analysis, however, response variability did not differ between TMS conditions (F_1,151_ = 1.0, p = .328, L.Ratio = 1.0) or between path types (F_1,151_ < 0.1, p = .932, L.Ratio < 0.1), and no interaction was found either (F_1,151_ = 0.1, p = .906, L.Ratio < 0.1). Similarly, in static trials, response variability did not differ between TMS conditions (t_21_ = 0.2, p = .869, *d* = 0.04).

## Discussion

In this study we used rTMS to perturb normal function of the precuneus. As a result, both working memory of spatial locations in static trials and the updating of object locations during simulated self-motion in dynamic trials were found to be impaired. Crucially, the deficit in spatial updating could not be explained by the working memory deficit alone, suggesting that the precuneus is essential (i) for working memory of object locations relative to the observer and (ii) for updating these locations within an egocentric map of space during self-motion. Together, these findings provide the first evidence for a causal role of the precuneus in spatial updating during simulated self-motion.

At first sight, alternative explanations of the observed stimulation effects seem conceivable. For example, rTMS over the precuneus may have simply interfered with the perception of optic flow based self-motion. Indeed, electrical stimulation of this structure can induce the sensation of translational self-motion^4^, and its activity during fMRI correlates with the subjective experience of self-motion^19^. However, the finding that memory in static trials, where no self-motion was simulated, was also affected by rTMS over the precuneus – even though this was only a trend –, argues against this alternative interpretation. Another alternative interpretation would state that the precuneus supports the preparation of motor responses (i.e., pointing), because the human precuneus is thought to contain a homologue of the monkey parietal reach region^20,21^. However, this explanation cannot account for the extra costs in response accuracy observed after rTMS in dynamic as opposed to static trials. Moreover, in our paradigm subjects did not have to perform a pointing response but used a joystick to continuously rotate their perspective instead.

We therefore propose that in the present study, rTMS over the precuneus interfered (i) with working memory representations for egocentric object vectors – in line with previous studies showing evidence for short- and long-term memory traces of egocentric space in medial parietal cortex^22,23^, and (ii) with the continuous, moment-to-moment updating of these vectors during self-motion. Spatial updating over short time scales and in an unfamiliar environment – as required here – has been suggested to operate on transient, egocentric representations, in which the relationship between each object and the observer is constantly updated as the observer moves^2^. This mechanism requires accurate processing of the continuous stream of incoming self-motion cues, which are known to reach the precuneus via direct projections from areas such as MST, VIP and 7a^24,25^.

When humans navigate in familiar environments or over longer durations and distances, however, they may mainly monitor changes in self-orientation and self-position by means of path integration, and locations of surrounding objects are stored in an enduring, allocentric map of space^26^. For updating in allocentric space, the entorhinal cortex rather than the precuneus is supposed to be essential due to its role in path integration^27^. In contrast, in our earlier study that used a comparable experimental setup but involved additional memory load manipulations^1^, it was found that increasing load also boosted the BOLD responses in the precuneus during spatial updating. This strongly suggests that the paradigms implemented here and in the earlier study were solved in egocentric space.

Finally, three limitations of the present study need to be mentioned. First, the effects of rTMS were presumably not limited to the precuneus but may have extended to neighboring regions of medial parietal cortex and beyond. However, by taking the findings of earlier fMRI studies into account^1^; the precuneus appears to be the brain region essential for spatial updating. Second, because we only provided visually simulated self-motion within an immersive virtual environment, participants were deprived of the vestibular and proprioceptive cues that contribute to the calculation of self-motion in everyday life. Therefore, in future studies, it will be important to apply rTMS over the precuneus before subjects freely move about in a virtual – or even a real – world scenario. This would allow for assessing whether the precuneus also plays a crucial role in spatial updating when multisensory self-motion cues are available. Third, future studies should rule out the possibility that inhibition of the precuneus interferes with the perception of movement per se, for example by implementing pointing responses to environmental objects remaining visible during the complete movement along different paths (compared to the present study, where the objects vanished before the movement onset).

## Materials and Methods

All methods were performed in accordance with the relevant guidelines and regulations^28,29^. The study was approved by the ethics committee of the Otto von Guericke University Magdeburg, Germany.

### Participants

Twenty-two healthy young participants (10 males, mean age 23.1 years, ranging from 19 to 30) were recruited among students of the Medical Faculty in Magdeburg by means of public bulletins. All subjects reported normal or corrected-to-normal vision, and all but three were right-handed. Exclusion criteria were auditory impairments (e.g., tinnitus) and a history of epileptic seizures. Participants received monetary compensation and gave written informed consent to participate in the experiment.

### Experimental setting and task

Participants performed a spatial updating task lasting for less than 20 minutes, in which they experienced passive self-motion within an immersive virtual environment projected onto a large, semi-circular screen (Fig. 1a) with a radius of 121 cm (380 cm in width and 196 cm in height, Arene Tech, Strasbourg, France). Participants sat on a comfortable chair positioned at the center of the half circle. The covered visual angle was 78 degrees vertically and 180 degrees horizontally.

The virtual environment was composed of a green plane containing white limited lifetime dots, each of which appeared at a random location for maximally 2 s before vanishing and reappearing at a different location (Fig. 1b,c; cf.^30^). When moving through the environment, the dots were the only source of information to estimate traveled distances and turns, but they could not be used as fixed landmarks.

In so-called *dynamic trials*, a target object was presented at a distance of 100 virtual meters (vm) at one of eight possible locations relative to the participant (Fig. 1b). After 2 s, the target object vanished by sinking below the ground, and the observer was passively moved along one of eight possible paths (cf. Fig. 1d). Paths consisted of a straight segment and a uniform curve, ranging from 60 to 120 degree rotation. The straight segment was 10 vm in length and the curved segment 30vm. All paths were traveled at a constant speed of 5 vm/s. The distribution of left- and rightward turns was kept constant.

After the movement was completed, a vertical black line appeared on the screen, and participants were asked to rotate with a joystick to align it with the remembered location of the target object (Fig. 1c). Responses were confirmed by pressing a joystick button. Between trials, the environment was occluded by a grey screen. Experimental stimuli were controlled by Vizard (v4.10.0005, WorldViz).

Theoretically, the interference effects induced by TMS could lead (i) to an increased bias in the pointing responses and/or (ii) to increased noise. A common approach to quantify noise is to measure the variability of behavioral responses across replications of a given trial type^1,26^. To implement this approach in the current study, we paired each path with only one particular target position (Fig. 1d). Because each path was repeated six times (see below), this approach allowed for testing whether TMS stimulation induced additional noise – quantified as the variability of the pointing error across replications of each path. Importantly, for each repetition, the speed of rotation was different (20, 25, 30, 35, 40 or 45 degrees per second; order randomized). This manipulation ensured that responses could not be based on timing strategies^31^, i.e., rotating for a certain duration rather than a certain angle.

To account for working memory effects independent of spatial updating performance, we also included 12 *static* trials. These trials did not present the observer with optic flow based passive motion, and they maintained their position and orientation for the same duration as during dynamic trials. Thus, each participant completed 60 trials in total. Eight practice trials were performed prior to the task.

### TMS application

The spatial updating task was performed in two experimental sessions (counterbalanced order). In the rTMS session, we applied a continuous theta-burst stimulation (cTBS) protocol centrally over the precuneus. Previous studies have shown that the inhibitory effect of cTBS lasts for up to 30 min after the stimulation^15–17^. Due to this off-line approach for TMS application, the experimental task was performed in the complete absence of any stimulation, which reduces the potential influence of unspecific effects^15,18,32^. In a control condition, we administered sham stimulation by turning the coil upside down so that the TMS impulses did not reach the brain. In addition, a sham noise generator (MagVenture, Hückelhoven, Germany) was used in both conditions. Due to this procedure, acoustic disturbance and vibrations of the coil were identical across both sessions. Sessions were conducted on two different, non-consecutive days (mean 5.64 days between sessions, ranging from 2 to 16 days).

Stimulation site was determined on the basis of spatial coordinates of the right precuneus as provided by^1^. As slightly different Talairach coordinates for the right precuneus were reported for the different experiments of that study, we averaged the values across experiments, resulting in the following coordinates: x = 5.33, y = −54.33, z = 47.33. These values were entered in a navigator system (Localite TMS Navigator, version 2.1.18), whose software enabled co-registration of the individual’s scalp surface via optical tracking supported by infrared marks (Polaris, NDI medical, Waterloo, Canada) and warped an MNI template brain to match the individual’s head. With that information the coil position and orientation on the scalp surface relative to the entered stimulation site coordinates were calculated by the system and monitored during stimulation.

TMS was controlled by a MagPro stimulator (X100 + MagOption, MagVenture, Hückelhoven, Germany), and pulses were delivered by a water-cooled figure-of-eight coil with an outer diameter of 75 mm (Cool B-65, MagVenture). We applied continuous theta burst stimulation according to previously described protocols^15,18^. Bursts containing three biphasic pulses (repeated at 30 Hz) were applied for 44 s at 6 Hz. Thus, one train of theta-burst stimulation consisted of 267 bursts (801 single pulses). The spatial updating task was performed immediately after TMS application.

Pulse intensity was individually set to 100% of the resting motor threshold (MT), which was defined as the lowest intensity capable to induce a motor evoked potential of 100µV (recorded from the right abductor pollicis brevis) in at least 50% of a series of ten single pulses applied to the left motor cortex. MT was assessed for both sessions separately. Mean pulse intensity for all subjects was 45.9% (ranging from 36% to 52%) of the maximal stimulator intensity.

### Statistical analysis

For each of the four path types we calculated the ‘updating angle’, defined as the change of the egocentric direction towards the target object before vs. after the passive movement along the path (cf. Fig 1e). This parameter quantifies the extent of spatial updating required for each path-target combination. Updating angle for path type 1 to 4 was 135°, 108°, 117° and 80°, respectively (cf. Fig 1d).

Our dependent measure was the signed pointing error, i.e., the deviation of the indicated response (in degree) from the egocentric target direction. For each trial, this calculation was performed irrespective of the direction of rotation, i.e., negative values indicated undershoot and positive values overshoot with respect to the correct response. Thus, the signed pointing error was always measured with respect to the direction the participant faced at the end of the outbound path. Negative values indicate an error towards this orientation (underreproduction of angle). Data were aggregated according to path type in dynamic trials (four symmetrical path pairs) and static trials.

Data were analyzed in R (version 3.0.2) with linear mixed effects models (2 × 4 factorial design), including the factors session (TMS vs. sham) and path type (4 levels). Subjects were included as a random factor. As static trials did not involve spatial updating and as they were less frequent, they were analyzed separately using t-tests. To assess the robustness of the inhibitory effect of cTBS, we performed an additional analysis including the first vs. the last third of trials as a further factor. To facilitate the interpretation of significant results, effect sizes are reported in terms of Cohen’s *d* (in case of t-tests) and the likelihood ratio (in case of linear mixed effects models), the latter representing the likelihood of a model including the current factor compared to a model without this factor.
