# Author Correction: Repetitive transcranial magnetic stimulation reveals a causal role of the human precuneus in spatial updating

**DOI:** 10.1038/s41598-018-31448-9

**Published:** 2018-09-07

**Authors:** Notger G. Müller, Martin Riemer, Lisa Brandt, Thomas Wolbers

**Affiliations:** German Centre for Neurodegenerative Diseases (DZNE), Center for Behavioral Brain Sciences (CBBS) & Medical Faculty at Otto von Guericke University, Leipziger Str. 44, 39120 Magdeburg, Germany

Correction to: *Scientific Reports* 10.1038/s41598-018-28487-7, published online 05 July 2018

This Article contains errors in Figure 1. The labelling in panel d is incorrect and panel e was omitted. The correct Figure 1 appears below.

**Figure 1:**
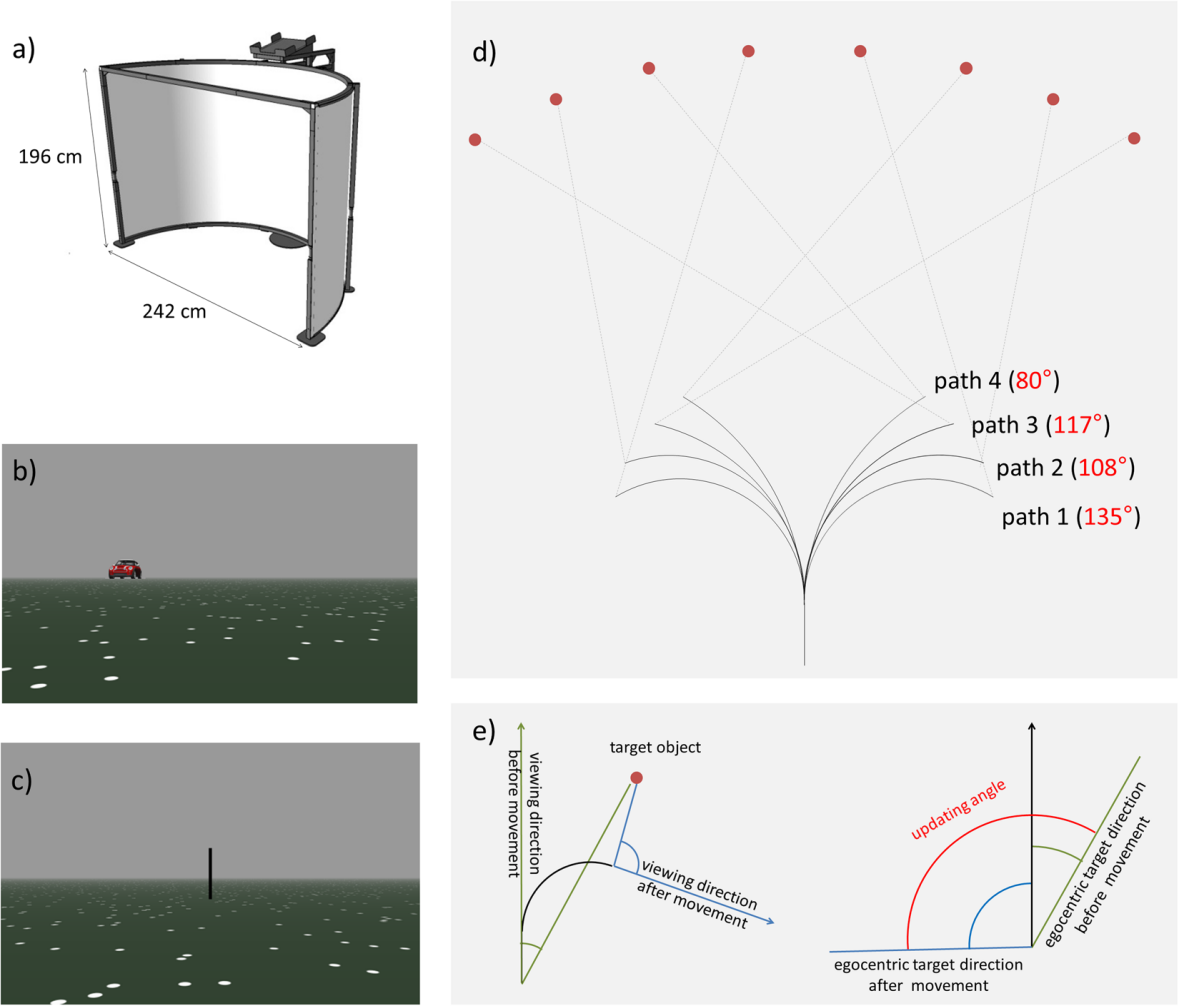
Experimental setup. (**a**) Circular screen used for the presentation of the virtual environment as shown in (**b**) and (**c**). (**d**) Schematic depiction of the eight different paths and their respective target locations. Numbers in red denote the updating angle associated with each path-target combination. (**e**) Updating angle was quantified by the angular difference of the egocentric direction towards the target object before (green angle) and after passive movement (blue angle). Copyright Information: (**a**) was provided for this publication by the manufacturer of the c-screen (Arene Tech, Strassbourg, France). (**b**,**c**) are screenshots from the actual experiment. (**d**,**e**) was drawn by one of the authors (MR).

